# Detroit Medical College

**Published:** 1871-11

**Authors:** 


### Detroit Medical College.

The following resolution of the Faculty of the Detroit Medical College removes one of the objections which have been urged against Spring College

Sessions, "That by this means students were enabled to obtain all their College instruction and to graduate within the short period of a few months."

REQUIREMENTS FOR GRADUATION.

"Diplomas will be conferred only once a year, at the close of the regular term.

Evidences of having studied medicine during a period of three years, and attended at least two courses of lectures, of which the last must have been in this institution, will be required of every candidate for graduation. *No student, however, will be received into the graduating class whose first course of lectures has been completed within six months of the beginning of the term.* This rule has been found necessary in order to prevent students from completing their whole college attendance in one year, a practice, the evils of which are too apparent to require discussion This rule will be rigidly adhered to under all circumstances"

